# Probability of misclassifying biological elements in surface waters

**DOI:** 10.1007/s10661-017-6368-6

**Published:** 2017-11-24

**Authors:** Małgorzata Loga, Anna Wierzchołowska-Dziedzic

**Affiliations:** 1 Faculty of Building Services, Hydro and Environmental Engineering, Warsaw University of Technology, Nowowiejska 20, 00-653 Warsaw, Poland; 2 The Polish National Energy Conservation Agency, Al. Jerozolimskie 65/79, 00-697 Warsaw, Poland

**Keywords:** Ecological status assessment, Biological indicators, Classification uncertainty, Misclassification, Monte-Carlo models

## Abstract

Measurement uncertainties are inherent to assessment of biological indices of water bodies. The effect of these uncertainties *on* the probability of misclassification of ecological status is the subject of this paper. Four Monte-Carlo (M-C) models were applied to simulate the occurrence of random errors in the measurements of metrics corresponding to four biological elements of surface waters: macrophytes, phytoplankton, phytobenthos, and benthic macroinvertebrates. Long series of error-prone measurement values of these metrics, generated by M-C models, were used to identify cases in which values of any of the four biological indices lay outside of the “true” water body class, i.e., outside the class assigned from the actual physical measurements. Fraction of such cases in the M-C generated series was used to estimate the probability of misclassification. The method is particularly useful for estimating the probability of misclassification of the ecological status of surface water bodies in the case of short sequences of measurements of biological indices. The results of the Monte-Carlo simulations show a relatively high sensitivity of this probability to measurement errors of the river macrophyte index (MIR) and high robustness to measurement errors of the benthic macroinvertebrate index (MMI). The proposed method of using Monte-Carlo models to estimate the probability of misclassification has significant potential for assessing the uncertainty of water body status reported to the EC by the EU member countries according to WFD. The method can be readily applied also in risk assessment of water management decisions before adopting the status dependent corrective actions.

## Introduction

It is in general expectation from the EU member countries to assess and report the status of water bodies within their boundaries *together* with a measure of uncertainty of the assessment, such as the confidence interval, precision, or probability of misclassification. The width of the confidence interval or the standardised precision of physico-chemical indices are commonly used measures of the uncertainty of water body (w.b.) status. Abundant measurements of these indices permit the calculation of statistical parameters for uncertainty measures with a high level of confidence. In special cases, the measurement series of physicochemical indices from multiple monitoring sites are combined into a single, larger data set. As a result, the corresponding statistical measures of the uncertainty of the w.b. status can be determined with a higher level of confidence. Unlike physicochemical parameters, which have been measured by all European water monitoring systems for more than 30 years, measurements of biological parameters in surface waters have only been subject to unified measurement protocols since the launching of the Water Framework Directive (WFD) monitoring programmes; in Poland, since 2006.

Typically, in *surveillance* monitoring, biological parameters are measured only once in each six-year cycle of water management planning (CIS 2003). Even in the case of *operational* monitoring, when measurements of these parameters are performed once per annum in a given w.b. for two consecutive years, combining the measurement series from several years does not allow the determination of one standard deviation value or stable confidence interval that would characterize a spread of measured values over longer periods. This is because of the inherent drift in aquatic ecosystems resulting from their reaction to the long-term anthropogenic changes in the catchment. As a result, *merged* series of measurement data of biological indices over a period of a dozen years cannot constitute the basis for estimation of the unique value of any chosen uncertainty measure. In particular, such data cannot be used to estimate the risks related to *corrective actions* undertaken in six-year cycles of water management.

Pioneering work concerning the sources of uncertainty in assessing w.b. status from biological sampling, and an assessment of errors in the standard methods of sampling for biological parameters have been undertaken by Clarke et al. (1996). These issues have also been addressed within the author’s subsequent studies Clarke and Hering (2006), Clarke (2013).

Field experiments designed to estimate uncertainty in the assessment of w.b. status based on biological measurements are usually carried out either by repeating measurements in a given w.b. several times by the same researcher (*repeatability*) or by performing measurements simultaneously by several researchers (*reproducibility*). This type of comparative research has been reported by various teams (Kolada et al. 2014; Kilroy et al. 2013; Szoszkiewicz et al. 2007). To measure the degree of spread in data obtained from multiple measurements of a biological parameter under the conditions of repeatability or reproducibility, the teams used the notion of *precision*. However, research of this type, being labour-intensive and costly, is usually performed for only a small number of repetitions (Szoszkiewicz et al. 2009; Bennett et al. 2011). Works of exceptionally numerous groups of experts are reported by Besse-Lototskaya et al. (2006) and Kahlert et al. (2012) who describe results of a pan-European diatom intercalibration exercise carried out within STAR project. As a consequence, the statistical estimators—the mean or standard deviation of the measurement data sets—obtained from such experiments are biased. When the number of repetitions is small, dynamic processes in the aquatic environment during the growing season and spatial heterogeneity of the water bodies may result in data sets in which the mean value is not representative of either the w.b. or the season. However, similarities between measurements taken in the same season in number of years, in contrast to measurements from different periods, have been observed by Lorenz and Clarke (2006) and interpreted as resulting from seasonal variability in the biological processes of the water bodies. Others, e.g., Šporka et al. (2006) and Johnson et al. (2012), have also pointed out the seasonal similarity in biological indices.

In this article, the overall effect of measurement errors of four biological components of water quality—macrophytes, phytoplankton, phytobenthos, and benthic macroinvertebrates—*on* the probability of misclassification of the ecological status of water bodies is examined without discussing the errors’ origins. Methods applied in monitoring of biological elements that have been adopted by Polish law and positively intercalibrated (EC 2013) are used in the article merely as the examples of general situation when biological data are scarce.

At the outset of the analysis, the WFD rule has been adopted that states that *the value* of a biological index *resulting from the measurement* made at some representative point of the w.b. uniquely identifies *the class of that water body in given year* in reference to a given index. To assess the probability of misclassification of the ecological status of a w.b., the spread of possible random measurement values of biological indices was modelled using the Monte-Carlo (M-C) method. Information on magnitudes of the biological measurement errors, provided by experts as well as the one presented in the reports from intercalibration exercises, was used to set parameters of the M-C models corresponding to particular biological elements.

## Data and methods

### Study area and measurement data

Data on the biological elements from the period 2006–2014, which served as the basis for the M-C simulations, were acquired from the river monitoring systems of the Voivodeship Inspectorate of Environment Protection of two Polish provinces—the Dolnośląskie and the Pomorskie voivodeships. The provinces are located within two geographical regions with distinct landscapes. The rivers in these regions represent a total of 20 out of 26 abiotic types of water bodies specified in the country. Table 1 presents the total number of water bodies in the two provinces for which index values of biological components measured at least once within the period 2006–2014, were available. Scarcity of the data is clearly visible.

**Table 1:** Number of water bodies with available index values of biological quality

	Macrophytes	Phytoplankton	Phytobenthos	Benthic macroinvertebrates
Dolnośląskie voivodeship	56	5	304	46
Pomorskie voivodeship	133	23	174	21

### Measurement errors and their simulation

When measuring a variable or parameter that is used for the determination of a biological index in the field or laboratory, like estimating the area of water covered by particular species of macrophytes or counting plankton cells by microscopic observations, the person performing the measurement practically never “hits” the parameter “actual value”. As the result the evaluated value of the index (which mathematically is a function of several measurable variables and/or parameters) only incidentally may adopt a value equal to the “actual value”. It is assumed from here onwards that “actual value” of index is equal to the expected value of this random function. It can be sufficiently well approximated from long series of its values evaluated from some directly, and possibly erroneously, measured metrics or parameters. As such long series of biological indices do not exist or, even if they would, they could not produce one meaningful long-term mean value. Therefore, it has been assumed pragmatically that “actual value” of each biological index is equal to its value calculated from the actual physical measurements of its corresponding parameters made at given w.b. at some “instant of time”. By assuming this definition of the index “actual value”, the WFD rule mentioned above allows for making the unique classification of the w.b. for some WFD-specified period of time. This assumption was realized by setting and running four M-C models simulating for each biological component departures from its index “actual value” that result from error-prone measurements of its parameters. Type of randomness and magnitudes of simulated measurement errors have been assessed by experts. The WFD-induced intercalibration exercises, that have been carried out in Poland for the purpose of incorporating indices of biological components into the surface water monitoring systems (Buffagni et al. 2006; Hutorowicz and Pasztaleniec 2014; Kolada et al. 2016), have provided the experimental basis for setting the M-C models of biological indices. The M-C simulations, that followed, have generated statistically meaningful sets of data used further in quantification of the effect of the measurement errors on the probability of misclassification of the water bodies.

M-C models were applied to avoid high costs associated with multiple physical measurements. They allowed for inexpensive computer simulations of the measurement procedures of the four biological indices of water quality: the river macrophyte index (MIR, Szoszkiewicz et al. 2002, 2009), the phytoplankton index (IFPL, Błachuta and Picińska-Fałtynowicz 2012; Mischke et al. 2011), the diatom index (IO, Błachuta and Picińska-Fałtynowicz 2010; Rimet and Bouchez 2012) and the multimetric benthic invertebrate index (MMI, Bis and Mikulec 2013; Lewin et al. 2013).

The occurrence of errors in the repetitive measurements (of whatever nature the errors could be) was simulated with the M-C models by generating series of the pseudo-random numbers with the assumed probability distribution. In all cases, the uniform distribution was assumed as a first approximation. The number of outcomes of the M-C *simulated* measurements for each biological index was sufficiently high to guarantee an unbiased estimate of the standard deviation from the index “actual value”.

### Monte-Carlo model for macrophytes

Estimation of the river macrophyte index (MIR) requires the identification of plant species within the selected river section as well as the estimation of the percentage of cover of the surface of the study area by particular species using a nine-point scale—so called, *cover index*.

When the “actual” percentage of cover in the study area is close to a threshold value for a given scale interval, the macrophyte cover index (estimated from the M-C simulated measurements) may be assigned to a neighbouring interval. Differences in the cover index of up to three points on the nine-point scale were encountered in some studies on macrophytes (Staniszewski et al. 2006).

It is assumed in this paper that (as a result of measuring errors) the value of the cover index can vary by one point on the scale, up or down, or stay unchanged, in relation to the actual measured value. By applying this assumption independently for each taxon, the range of values that characterise the MIR in a given year can be assessed.

It was shown through numerical experimentation that performing 20,000 repetitions of the MIR calculations using degree of cover values with randomly generated errors was sufficient to obtain a stable estimate of the standard deviation and the stable probability of misclassification of the w.b. status based on this biological index.

### Monte-Carlo model for phytoplankton

In Poland, the assessment of the ecological status of water bodies based on the quantity of phytoplankton is performed by calculating the multimetric phytoplankton index (IFPL) which is the arithmetic mean of the trophic index (TI) and the chlorophyll index (CH) (Błachuta and Picińska-Fałtynowicz 2012; Rimet and Bouchez 2012).

The measured values used for the evaluation of the IFPL are the biovolumes of the indicator taxa and the *chl-a* concentration. In this study, it has been tested that 10% distortion of cell’s original linear dimensions leads to 20% biovolume error.

According to data from the US (http\\diatom.ansp.org\autoecology) errors in phytoplankton biovolume can reach even 300% and errors exceeding 30% are very common (for almost 75% of all analyzed species). In this paper, an approximation of biovolume error of 30% for phytoplankton datasets was adopted.

In the case of *chl-a* concentration, the accuracy of the CH module is high. However, because the total error is also determined based on the sampling error and the non-uniform character of the sample, this study adopted an error of 10% *chl-a* measurement. From the criterion of the number of distorted measurements necessary to obtain a stable standard deviation of IFPL, it was determined that 1000 is a sufficient number of generated IFPL values.

### Monte-Carlo model for phytobenthos

The analysis of the ecological status of surface waters based on phytobenthos is performed in Poland by the determination of the multimetric diatom index (IO) (Błachuta and Picińska-Fałtynowicz 2010, Kelly et al. 2008). To determine components of the IO, the estimate of the abundance of individuals belonging to a given taxon can be considered as a *simple measurement*. The effect of the error in the determination of the abundance of individuals belonging to a given taxon on the value of the diatom index was simulated using the M-C model. Random distortions of order 15–20% (J. Błachuta—personal comm.) were introduced into the *measured* values of abundance of various taxa, and the respective values of the IO were determined for the distorted values.

One thousand measurements of the IO were simulated for each of the analyzed w.b. Each time, an independent distortion of the abundance of individuals of each species at a level of − 15, 0, or + 15% was introduced. One thousand M-C generated “measurements” was sufficient to stabilize the standard deviation of the IO.

### Monte-Carlo model for macrozoobenthos

In Poland, the assessment of the status of water bodies based on macroinvertebrates employs the benthic invertebrate index (MMI). This index is determined from the multimetric intercalibration index (ICMI)—the arithmetic mean of normalized values of several metrics (Bis and Mikulec 2013, Lewin et al. 2013).

Based on the expert opinion (J. Błachuta, S. Jusik) and information from the literature (Gobeyn et al. 2016), the analysis of components in the MMI formula has shown that the probability of a simple measurement error involving the underestimation or overestimation of the abundance of individuals in particular taxonomic groups is considerably higher than the probability of incorrect determination of a taxon. It has been suggested by the experts that the abundance measurement error is characterized by the uniform distribution with a statistical dispersion of approximately 20–25%. It was also established that stabilization of the standard deviation for distorted values of the MMI occurs at 1000 repetitions.

## Simulation results

In the final step of the analysis, the values of biological indices that had been generated by the M-C models, were assigned to the corresponding biotic classes of water quality according to the currently binding regulations. Values of the index calculated from sets of randomly disturbed measurements can indicate the same or another class as compared to the one assigned from the undistorted measurements.

**Fig. 1:**
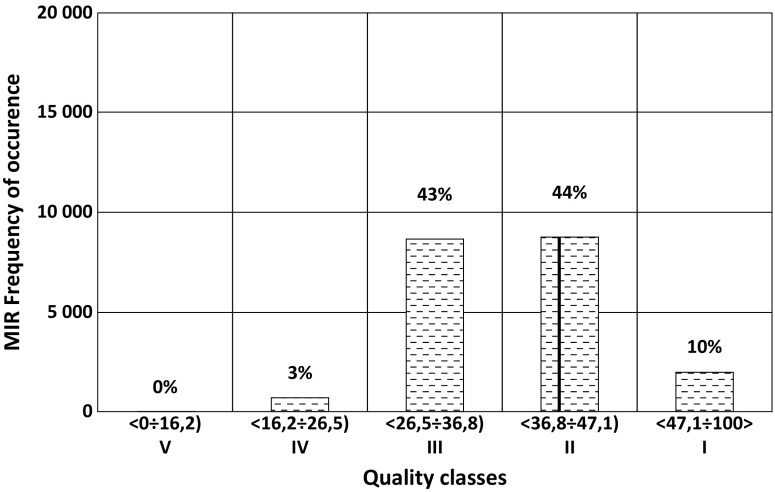
Random measurement distortions of MIR in the year 2010 showing the probability of classifying a given w.b. (Węgiermuca River) into more than one class. The numbers on top of the bars specify the percentage of cases in which values of the index have fallen into the classes. The vertical line indicates actual value of MIR from the year 2010

**Fig. 2:**
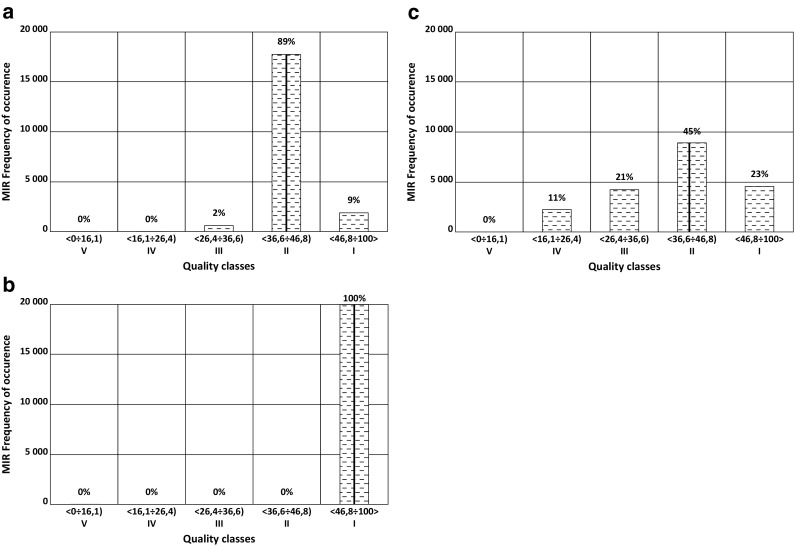
MIR index in years 2008, 2010, and 2012 (left to right) together with the MIR distributions resulting from random distortions of the measurements (for Nysa Łuzycka River). The numbers above the bars specify the percentage of cases in which values of the index have fallen into particular classes. The vertical line indicates actual value of MIR made in each year

**Fig. 3:**
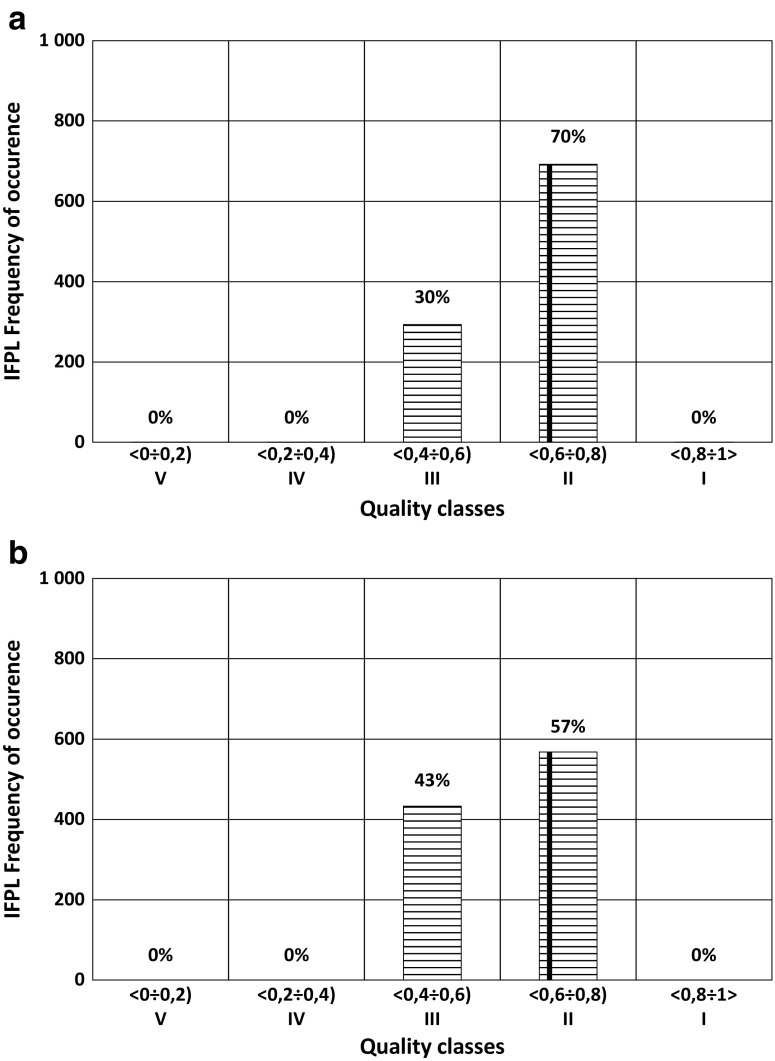
IFPL for the Bychowska Struga River. **a** Distortion of biovolume by 10% and chlorophyll concentration by 20%. **b** Distortion of biovolume by 30% and chlorophyll concentration by 10%. The vertical line indicates actual value of IFPL

**Fig. 4:**
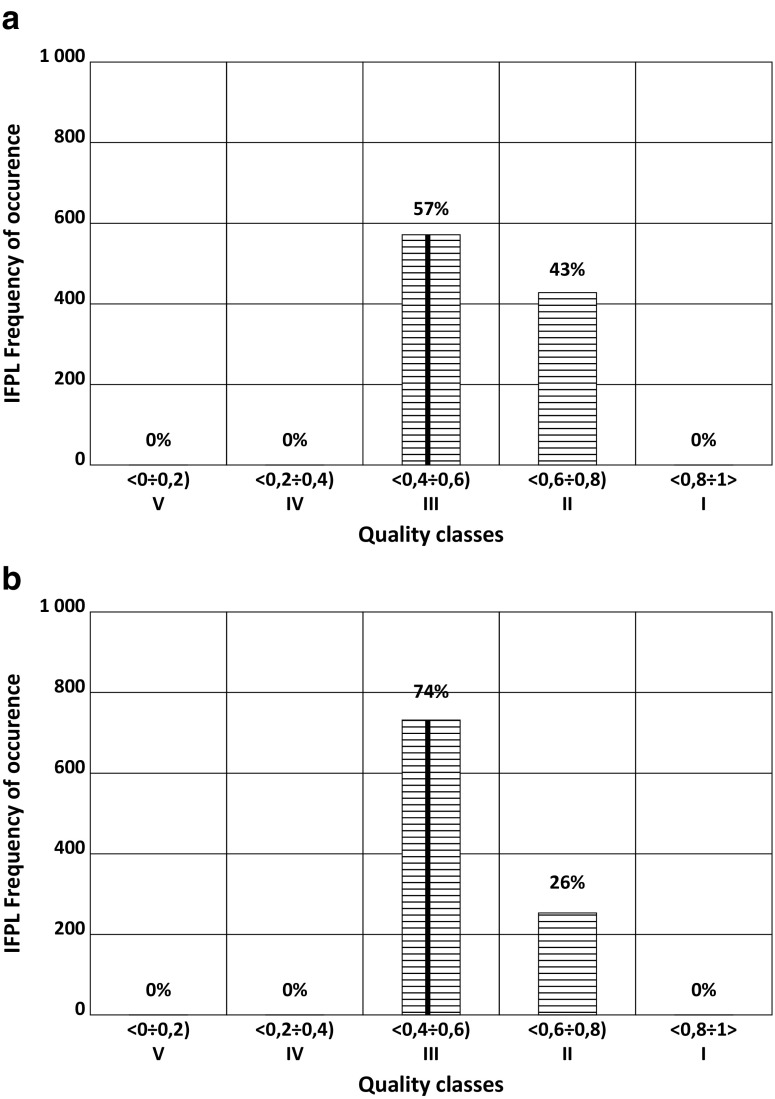
IFPL for the Odra River. **a** Distortion of biovolume by 10% and chlorophyll concentration by 20%. **b** distortion of biovolume by 30% and chlorophyll concentration by 10%. The vertical line indicates actual value of IFPL

**Fig. 5:**
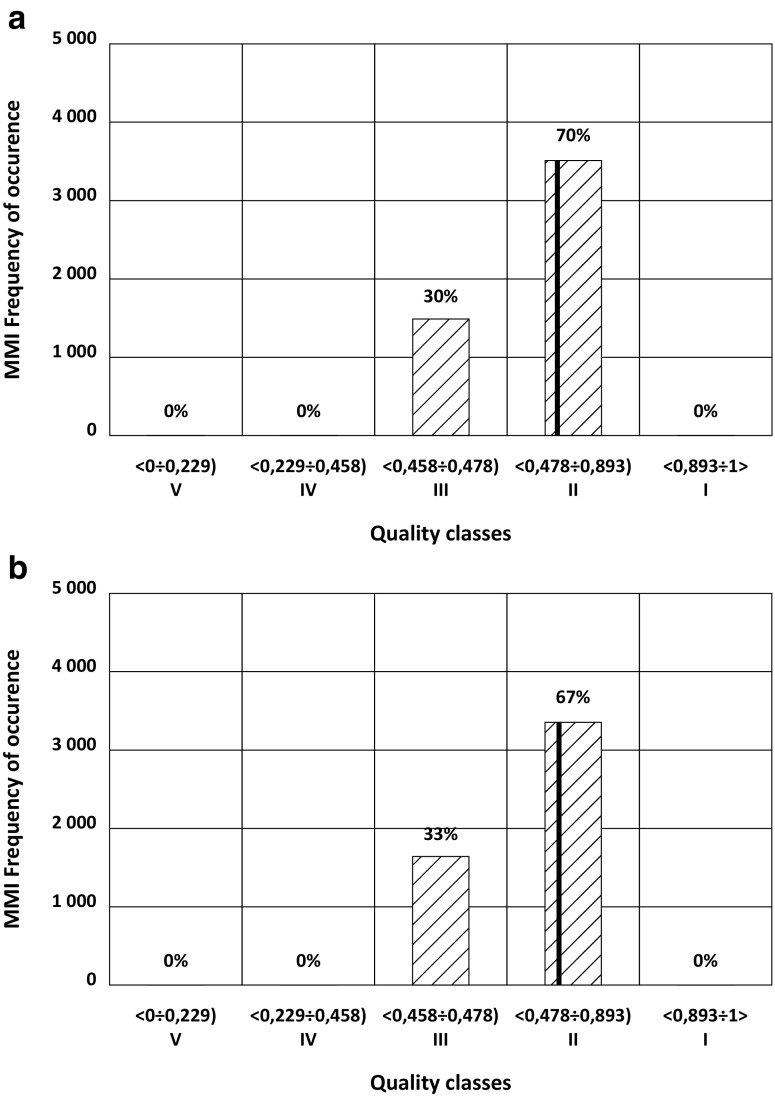
Classification based on MMI of Kamienica River in 2009 for two different values of measurement error in the number of individuals of macroinvertebrates: **a** 20 and **b** 25%. The vertical line indicates actual value of MMI from the year 2009

**Fig. 6:**
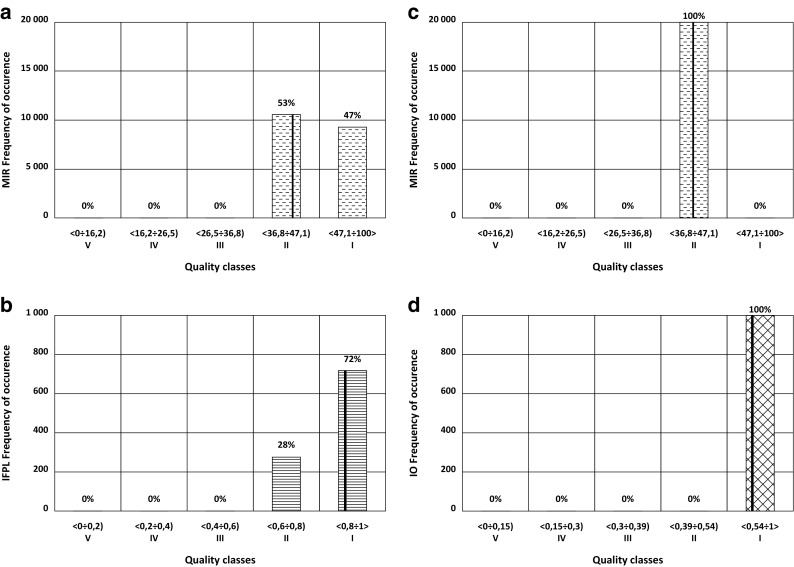
History of classification for Brda River from 2009 to 2014. **a** MIR in 2009, **b** IFPL in 2011, **c** MIR in 2012, and **d** IO in 2014. The overall assessment should be high instead of good due to the very high probability of high status of 47% in 2009. The vertical line indicates actual value of indices

The cases in which a distorted measurement leads to an assignment to another class is defined as a *misclassification*. Based on the number of occurrences of such cases, it is possible to estimate the probability of misclassification (PoM). Figures 1, 2, 3, 4, 5, and 6 present the results of the M-C simulations for various indices in terms of the frequency with which generated values of the indices occur. The surface water status *classes* indicated in the figures constitute the basis for the assessment of the ecological status of water bodies in Poland; they correspond to binding regulations in the country and are in line with EC decision (EC 2013).

### Misclassification related to macrophytes

In the majority of cases, the generated MIR index with the *cover index* distorted by one point (on the nine-point scale) caused a change in the w.b. status classification by one class in comparison with the original assessment. There were also cases in which the distortion of the cover coefficient resulted in a value of the MIR that changed the assessment of the w.b. status by two classes. Such an example is presented in Fig. 1. In this case, a class lower than that resulting from the undistorted MIR value is assigned almost as frequently as the original class. The two extreme classes (high and poor status) are assigned by a relatively lower but still considerable number of MIR values. In the example presented in Fig. 1, the probability of classification of a w.b. based on the macrophyte index to a status worse than *good* (i.e., to moderate or poor) is 46%, whereas the probability of classification of a w.b. to *high* status is 10%.

The Monte-Carlo simulations, such as those presented in Figs. 1 and 2, result in occurrence of various classes corresponding to (disturbed) measurements of MIR metrics. Importantly, the PoM can be assessed as the fraction of the cases in which the w.b. class differs from the real MIR, i.e., the MIR resulting from undistorted measurements. In the example presented in Fig. 1, the PoM is quite high: 56%.

As shown in Fig. 2, distortions of the measurements can cause incorrect assessment of the biological index value and thus lead to erroneous assignment of the w.b. class. Should one omit the year 2010, *good* status is maintained in 2012 (despite of decrease in the MIR in comparison with 2008) with the PoM higher though.

The probability of a status *above good* is higher in 2012 than in 2008 (the probability of *high* status is 23%), but the probability of classification to a status below *good* is also higher (33%).

The results of the classification of water bodies in the two voivodeships based on the MIR index calculated from distorted measurements of the *cover index* are presented in Table 2.

**Table 2:** Summary of the classification of water bodies in selected voivodeships based on measurements of the MIR index calculated from distorted measurements of the *cover index*

Effect of error in the measurement of the cover index	Dolnośląskie voivodeship (% of w.b.)	Pomorskie voivodeship (% of w.b.)	Total contribution (%)
Probability of a change in classification of the w.b. by one class	51.7	63.9	60.3
Probability of a change in classification of the w.b. by more than one class	30.4	15.8	20.1
No change in classification of the w.b.	17.9	20.3	19.6

### Misclassification related to phytoplankton

To judge which of the measurement errors—error in the measurement of chlorophyll concentrations or incorrect determination of biovolume—causes greater change in the value of the phytoplankton index IFPL, a number of simulations were performed in which distortions were introduced in pairs. The M-C model was used to generate simultaneous occurrences of these two types of errors; first, the chlorophyll concentration was distorted by 20% and the biovolume by 10% and second, the chlorophyll concentration distortion was lowered by 10% and error in the biovolume was increased by 30% compared with the measured values.

The results suggest that in the case of these particular combinations of distortions, similar changes in w.b. classification are observed; e.g., distorting the biovolume by 10% and the chlorophyll concentration by 20% (Figs. 3a and 4a) had a smaller effect on IFPL than distorting the biovolume by 30% and the chlorophyll concentration by 10% (Figs. 3b and 4b).

In the cases presented in Fig. 3a, b, the phytoplankton index indicates *good* status of the two water bodies. In the first case, however, the probability of an incorrect assignment of the w.b. status is 30%, whereas in the second case it is greater than 40%.

In the cases presented in Fig. 4a, b, a high PoM occurs: 43 and 26%, respectively, although the value of the IFPL measurement is 0.48, which is in the middle of the class interval for *moderate* status.

When disturbing the measured components of IFPL, a change in class was observed in 7 out of 27 water bodies (i.e., in 26% of all analyzed w.b.). The majority of cases corresponding to the incorrect class assessment based on IFPL values were related to the situations where value of the index was occurring near the boundary between classes.

It was also observed that changes in IFPL values in the case of measurement error in the biovolume of particular taxa were dependent on the species composition of phytoplankton and their weighting factors.

### Misclassification related to phytobenthos

Changes in the w.b. class assessed for the diatom index (IO) frequently occurred when the measured index value was close to the class boundary. However, in most of these cases, the probability of assigning a class consistent with the class calculated from the undisturbed measurement was higher than the PoM. Cases also occurred in which the probabilities for both classes were virtually identical.

The simulation results indicate that 27% of the studied water bodies could be misclassified when classification was based on phytobenthos. However, PoM probability is rather low in all these cases. This can be explained by the structure of the IO index (three components) and the weights ascribed to particular species indicators. For this reason, measurements of phytobenthos show high credibility in the assessment of the ecological status of water bodies.

### Misclassification related to macroinvertebrates

As with other biological indices, the simulation of error-prone measurements of the abundance of species in particular taxons of benthic macroinvertebrates with the M-C model showed that the standard deviation of the MMI index was stable after 1000 repetitions. An increase of the distortion of the measurements from 20 to 25% (Fig. 5) did not change the classification results.

The analysis of the robustness of the benthic macroinvertebrates index to measurement distortions was carried out by analysing measurements from 67 water bodies. This sample was considerably smaller than for macrophytes and phytobenthos despite the inclusion of all historical measurement data available from the two voivodeships.

Adding the simulated error into the measured abundance of the organisms resulted in a change of the ecological status from the actual status in 5 out of 67 water bodies (7% of cases). In the remaining 93% of water bodies, high robustness of the MMI index to measurement errors was observed. Water bodies in which a distortion of measurement values affected their status were those for which the MMI (actual) value was near the threshold value of the class characterizing the status of water quality based on macrozoobenthos.

## Discussion

Because of slow natural response of biological indices to changing environment conditions, the sampling frequency of biological parameters in all European water monitoring systems has been chosen low. Consequently, the statistical uncertainty in the assessed ecological status of water bodies based on measurements of biological parameters can be considerably higher than the status uncertainty based on the frequently measured physicochemical indices. By requiring to determine the status (or class) of water bodies from biological indices, the Water Framework Directive causes unintentionally an increase of risk in water management decisions based on the WFD. This article has presented an essential part of the risk assessment within the domain of water management—the estimation of the probability of misclassification of water bodies.

The M-C model applied in this study has introduced a considerable simplification to the PoM analysis as compared to the factorial design of experiments. For instance, to calculate *all* possible values of the MIR index in accordance with the *three-level full factorial design* procedure, when M species of macrophytes have been identified within a section of the river, one needs to perform *N* = 3^M^ evaluations of the index. In the case of *M* = 20 species identified in the field, this amounts to almost 3.5 million evaluations. For other biological water quality elements, number of their taxons can be much higher than 20 and therefore following this factorial design procedure could have been lead to even more problematic number of repetition of index calculations.

As exemplified above, number *N* could be an excessive number from the standpoint of this study objective. Simplification introduced in this research by the M-C simulation was based on the premise that it is sufficient to consider a much smaller number of distorted measurements and relevant calculations of the MIR or other index to guarantee a stable estimate of the spread of index value. For instance, in the case of phytobenthos it was enough to simulate distortions of the abundance of each species by running the M-C model 1000 times for the three-point distribution at levels − 15, 0, or + 15% each having the same probability of occurrence. Naturally, the three-point distribution of distortions in phytobenthos measurements could be substituted with some continuous or mixed discrete-continuous distributions.

Recently, the M-C model assuming a normal distribution of the measurement error has been applied to analyze the uncertainty in the w.b. status assessed from measurements of benthic macroinvertebrates (Gobeyn et al. 2016). However, the data sets used by Gobeyn and his co-workers were many times larger than the sets considered in this paper.

It needs to be remarked that the approach presented in this paper for simulating occurrence of errors in estimation of taxa abundance or percentage of water surface covered with given taxa, does not take into account wrong identification of species. Ambiguous recognition of diatoms, especially when they represent significantly different sensitivity to environmental conditions (Kahlert et al. 2012) or cases of overlooked taxa can even more influence the values of the indexes than changes in abundance.

The errors in macrophytes recognition and the corresponding uncertainties were studied by Wiedeker et al. (2015). These authors introduced the so-called confusion matrix for macrophytes to assess the uncertainty of a IBMR index applied in France. Also in the research of Wiederker sets of data were many times larger than the sets analyzed in this paper.

In the context of w.b. classification, an additional problem arises when applying the OOAO (One Out All Out) rule suggested by CIS (2005). With regard to biological indices the discussion concerning OOAO issue is still on (Langhans et al. 2014). An example of Brda River (Fig. 6) alternately shows *good* status based on MIR in 2009, high status in 2011 based on IFPL, *good* status based on MIR in 2012, and high status based on IO in 2014. When classifying this water body in 2014, the *good* status based on the macrophyte index was assigned. However, given the approximate value of the MIR index in 2009 and the very high probability of high status as well as two high assessments suggested by the diatom and phytoplankton indices, it appears that the classification of the Brda River in 2014 as having high status is more accurate.

The monitoring data used in this analysis represents a limited dataset. This is because biological elements measurement data originated from rivers located only in two geographical regions and the available series of data were very short. Given the encouraging results obtained from estimation of the probability of misclassification of the w.b. status despite the limited data, the proposed method could be easily expanded for rivers with scarce biological data. The proposed method for determining the probability of w.b. status misclassification based on distorted measurements of biological parameters is also applicable when considering an assessment of ecological status from uncertain measurements of the physicochemical water quality elements. With regard to these elements, the problem of scarcity of measurement data does not arise because the corresponding data have been gathered by all EU countries already for several decades.

The subject of this study—impact of distortions and measurement errors in biological parameters on the classification of water bodies, is not only of scientific interest but it has also practical implications.

The presented method of quantifying the PoM of a water bodies based on biological elements, also provides the basis for estimating the risk associated with a spectrum of decisions concerning the corrective measures in the catchment which are to be considered in the situations when w.b. status is uncertain and its status may be assessed falsely.

Instances in which the index value is close to the threshold between *good* and *moderate* status classes are particularly important because of their consequences for water management and water protection decisions. In the majority of cases, this threshold determines whether the environmental objective is met.

Further research and analyses are planned on the development of an integrated methodology for assessing the probability of misclassification of the status of water bodies based on error-prone measurements of both physicochemical and biological elements.

The integration has been already initiated by general concept of hierarchical approach proposed by M. Loga (2012). It assumes the propagation of elementary measurement errors through a hierarchical structure of procedures that are used for determining the w.b. status in accordance with the WFD.

## Conclusions

The Monte-Carlo models, that are applied in science for simulating all kinds of random phenomena, have been successfully used in this research for modelling of uncertainties accompanying monitoring of surface waters.

The proposed M-C models allow for assessment of the probability of misclassification of the biological indices relevant for assessment of ecological status of water bodies in cases where only short series of measurements of biological elements are available. Therefore, it could be of special interest for member states of the European Union, which have only recently implemented procedures for monitoring these elements in surface waters in accordance with the Water Framework Directive. In particular, the M-C simulations that have been carried out in this research, indicate a relatively low resistance of the macrophyte index (MIR) and high resistance of the phytobenthos diatom index (IO) and benthic macroinvertebrates index (MMI) to the measurement errors.

Also, the OOAO rule, as a part of water body classification, can be integrated with the proposed method of assessing the probability of misclassification, allowing for deviation from the rule in certain cases. However, the statement regarding possible modifications of the mandatory OOAO rule should be tested on larger datasets.
